# Structural Features of Antibody-Peptide Recognition

**DOI:** 10.3389/fimmu.2022.910367

**Published:** 2022-07-07

**Authors:** Jessica H. Lee, Rui Yin, Gilad Ofek, Brian G. Pierce

**Affiliations:** ^1^Department of Cell Biology and Molecular Genetics, University of Maryland, College Park, MD, United States; ^2^University of Maryland Institute for Bioscience and Biotechnology Research, Rockville, MD, United States; ^3^University of Maryland Marlene and Stewart Greenebaum Comprehensive Cancer Center, Baltimore, MD, United States

**Keywords:** peptide, antibody, linear epitope, structure, immunology

## Abstract

Antibody recognition of antigens is a critical element of adaptive immunity. One key class of antibody-antigen complexes is comprised of antibodies targeting linear epitopes of proteins, which in some cases are conserved elements of viruses and pathogens of relevance for vaccine design and immunotherapy. Here we report a detailed analysis of the structural and interface features of this class of complexes, based on a set of nearly 200 nonredundant high resolution antibody-peptide complex structures that were assembled from the Protein Data Bank. We found that antibody-bound peptides adopt a broad range of conformations, often displaying limited secondary structure, and that the same peptide sequence bound by different antibodies can in many cases exhibit varying conformations. Propensities of contacts with antibody loops and extent of antibody binding conformational changes were found to be broadly similar to those for antibodies in complex with larger protein antigens. However, antibody-peptide interfaces showed lower buried surface areas and fewer hydrogen bonds than antibody-protein antigen complexes, while calculated binding energy per buried interface area was found to be higher on average for antibody-peptide interfaces, likely due in part to a greater proportion of buried hydrophobic residues and higher shape complementarity. This dataset and these observations can be of use for future studies focused on this class of interactions, including predictive computational modeling efforts and the design of antibodies or epitope-based vaccine immunogens.

## Introduction

Antigen recognition by antibodies is a key component of immunity in humans and other vertebrates. Antibodies are highly diverse in sequence, and are able to recognize a vast array of foreign antigens, including proteins, peptides, non-protein molecules, and combinations thereof (1). High resolution structural characterization of antibody-antigen interactions can yield insights into the basis of antibody breadth and protection (2–4), and can enable development of methods to predict antibody epitopes (5, 6) or model antibody-antigen complexes (7), as well as structure-based antibody (8, 9) and immunogen (10, 11) design efforts.

Antibody recognition of linear peptide epitopes is a highly important class of antibody-antigen complexes. Such complexes include broadly neutralizing antibodies that target viruses such as hepatitis C virus (HCV) and coronaviruses (12–14), as well as immunotherapeutic antibodies targeting host proteins (15), and structures of those complexes have led to immunogen designs for HCV (16), human immunodeficiency virus (HIV) (17), and respiratory syncytial virus (RSV) (18). Furthermore, antibody-peptide interactions are the basis for many commonly used protein purification and detection systems involving sequence tags, including Myc, HA, and FLAG tag systems (19). Previous studies have presented surveys and databases of protein-peptide complex structures (20, 21), and others have assessed structures of general antibody-antigen interfaces (1, 22, 23), providing major insights into these interactions. One study, published in the 1990’s, analyzed structures of six antibody-peptide complex structures that were available at the time (24). These studies have included either a limited or no representation of antibody-peptide complexes, leaving open the question of whether antibody-peptide complexes exhibit shared or distinct binding strategies and interface features in comparison with antibody-protein antigen complexes. One study from approximately five years ago did compare antibody-peptide interfaces with antibody-protein interfaces and other protein-protein interfaces, but the analysis included a limited set of features (hydrogen bonds and shape complementarity) (25).

Here we present a focused and updated analysis of antibody-peptide interfaces and their key features, including interface size, epitope secondary structure, antibody flexibility and epitope flexibility. While in some cases there are broad similarities between antibody-peptide and antibody-protein antigen interfaces, we also identified notable differences, and we also found a lack of regular secondary structure in many epitopes, as well as several cases of pronounced epitope plasticity. Collectively, our observations and structural dataset can inform future predictive modeling algorithms focused on antibody-peptide complexes, as with previously developed methods that perform modeling of antibody-protein antigen complexes (26, 27) or general protein-peptide complexes (28–30).

## Methods

### Dataset Assembly

A list of all experimentally determined antibody-peptide complexes available in the Protein Data Bank (PDB) (31) was downloaded from the SAbDab database (32) in December 2021. That set was then filtered to retain only structures with 3.0 Å resolution or better, structures without missing peptide residues (except for missing N-terminus and/or C-terminus residues), structures containing resolved peptide antigens of lengths between 5 and 20 residues, and only structures containing heavy-light chain antibodies (no single-chain nanobodies). A nonredundancy filter of 90% sequence identity by heavy chain variable domain (V domain) or 90% sequence identity for both V domains was used to remove structures with identical or highly related antibodies. To avoid inclusion of interactions mediated in part by non-protein atoms, all structures with non-water HETATM molecules within 4.5 Å of any atoms in the antigen and antibody were removed. ACE and NH2 atoms (corresponding to acetylation and amidation, respectively), which are commonly added to the termini of synthetic peptides to avoid non-native terminal charges, were not included for that filtering. The full antibody-peptide complex set (N=188) was filtered to a “nr_epitope” set (N=121) by agglomeratively grouping together pairs or sets of complexes that contain peptide sequences that match, or that have the smaller sequence contained in the larger peptide sequence, based on peptide residues present in the structures. One representative per set of paired or grouped complexes was selected for the “nr_epitope” set, based on structural resolution.

Unbound antibody structures matching the antibodies in antibody-peptide complexes were identified in the PDB through an adaptation of the program previously to identify unbound structures for Protein Docking Benchmark 5.5 (7). Initially identified candidate unbound antibody structures were filtered by resolution (3.0 Å or better) and lack of missing or mutated residues for antibody residues proximal to the peptide in the peptide-bound complex structures. As with Docking Benchmark 5.5, antibody interface residues were defined as those within 10 Å of the binding partner (i.e. the peptide) in the complex structure.

### Structural Analysis

Peptide residue secondary structure analysis was performed using the DSSP program (33). Peptide structure classifications were assigned as Helix [> 50% residues assigned α helix by DSSP, as used by Trellet et al. (30)], Coil (> 60% residues with no assigned DSSP secondary structure, “C”), Hairpin (or hairpin-like; 2 or more extended, “E”, residues, followed by two or more turn-like residues, “T”, “S”, or “C”, followed by two or more extended, “E”, residues), or Other. Molecular solvent accessible surface area (SASA) values for interface buried surface area (ΔSASA) calculations were calculated using the NACCESS program (34), by subtracting the complex SASA from the sum of the individually calculated antibody and peptide SASA values, as performed previously for complexes in the protein-protein docking benchmark (7).

Interface hydrogen bonds were calculated using the hbplus program (35), and antibody-peptide and antibody-antigen interface atomic contacts were calculated using a 4.5 Å cutoff distance between non-hydrogen atoms. Hydrophobic atom contact calculations were performed using side chain atoms of hydrophobic residues Trp, Tyr, Phe, Met, Ile, Leu, and Val; side chain atoms of those residues were grouped together in a previous analysis of transient protein-protein complex structures and the resultant IFACE statistical potential (Atom Types 6, 7, and 10) (36). Structure-based calculation of binding energies (ΔG) was performed using Rosetta’s “score” executable (weekly release 2020.25) and the Rosetta Energy Function 2015 (REF15) energy function (37), as performed previously (7). Before complex structures were processed in Rosetta, they were pre-processed with Rosetta’s FastRelax protocol to perform constrained minimization and remove spurious unfavorable geometries that would bias the Rosetta calculations. Flags used for FastRelax (“relax” executable) were:

-ignore_unrecognized_res
-relax:constrain_relax_to_start_coords
-relax:coord_constrain_sidechains
-relax:ramp_constraints false
-ex1
-ex2aro
-no_optH false
-flip_HNQ
-renumber_pdb F
-nstruct 1

The Rosetta InterfaceAnalyzer protocol was used to calculate the hydrophobic ΔSASA and shape complementarity (S_c_) interface features, after pre-processing with FastRelax as noted above. The S_c_ calculation in Rosetta was based on the Lawrence and Colman method (38).

For analysis of complementarity determining regions (CDR) loop conformational changes and antigen contacts, antibody variable domains were re-numbered using the ANARCI tool (39) based on the AHo antibody numbering system (40). CDR loops for both heavy and light chains were defined as residues 24-42 (CDR1), 57-76 (CDR2), and 107-138 (CDR3). These correspond to Kabat residue numbers 23-35 (CDRH1), 50-65 (CDRH2), 93-102 (CDRH3), 24-34 (CDRL1), 49-60 (CDRL2), and 89-97 (CDRL3). Root mean square distance calculations for antibody CDR, peptide conformations, and antibody-peptide interface residues were performed using backbone atoms and the ProFit program (41), and calculation of CDR loop binding RMSDs were performed after superposition of the corresponding full unbound and bound variable domain structures using FAST (42).

### Figures and Statistical Analyses

Figures of data were generated using R (r-project.org) and ggplot2 (43), as well as gnuplot (gnuplot.info), and figures of molecular structures were generated using PyMOL (Schrodinger, LLC). All statistical comparisons were performed using the Wilcoxon rank-sum test in R.

## Results

### Antibody-Bound Peptide Structural Features

**Figure 1:**
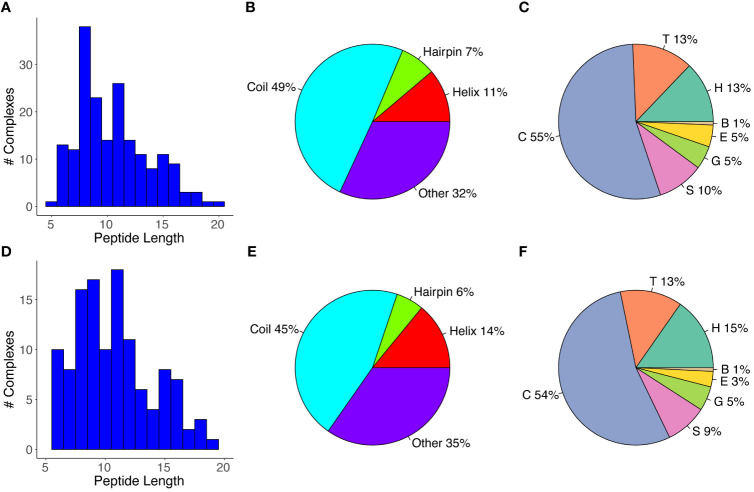
Antibody-bound peptide lengths and secondary structures. **(A)** A histogram of the peptide residue lengths, **(B)** Peptide structural classes, and **(C)** Peptide residue DSSP structural classes are shown for the full set of antibody-peptide complexes (N = 188). The **(D)** Peptide lengths, **(E)** Peptide structural classes, and **(F)** Peptide residue structural classes are also shown for the “nr_epitope” complex set (N = 121), which corresponds to the full set of complexes filtered by grouping pairs or sets of complexes with matching peptide sequences and retaining one representative per pair/group. Peptide structural classes **(B, E)** were defined based on DSSP (33) residue-level secondary structure assignments, as noted in the Methods, and peptide residue secondary structure classes **(C, F)** correspond to DSSP-assigned classes. Classes are α-helix (“H”), β-bridge (“B”), extended strand (“E”), 3_10_ helix (“G”), turn (“T”), bend (“S”), and no regular secondary structure (loop/irregular; “C”).

**Figure 2:**
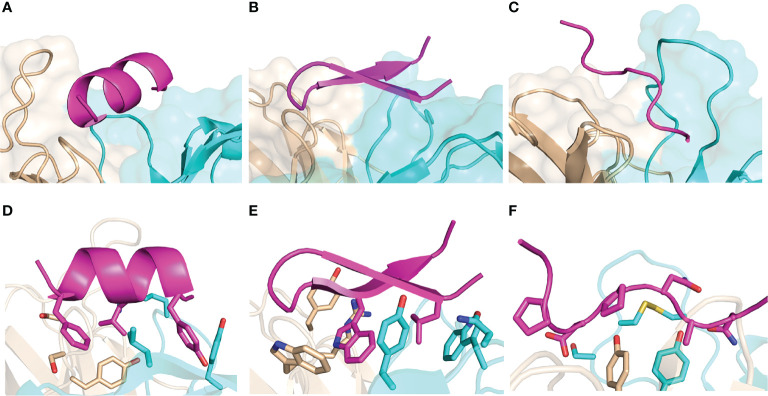
Examples of peptide-antibody complexes and structural classes. **(A, D)** SARS-CoV-2 spike stem in complex with antibody B6 (PDB code 7M53; Helix peptide), **(B, E)** HCV E2 peptide in complex with antibody HCV1 (PDB code 4DGY; Hairpin peptide), **(C, F)** P. falciparum circumsporozoite protein junctional epitope in complex with mAb668 (PDB code 6PBV; Coil peptide). Antibodies are shown in cyan (heavy chain) and tan (light chain), and peptides are magenta. **(D–F)** show selected interface side chains as sticks, with oxygen atoms red, nitrogen atoms red, and sulfur atoms yellow.

We assembled a set of antibody-peptide complex structures from the PDB based on nonredundancy, structural quality, and other criteria, as detailed in the Methods, leading to a total of 188 antibody-peptide complexes (**Table S1**). These structures include antibodies in Fab (fragment antigen-binding) and scFv (single chain variable fragment) formats, and they have resolutions ranging from 1.17-3.0 Å (median: 2.04 Å). A summary of the of the observed antibody-bound peptide lengths and secondary structures is shown in **Figure 1**. Lengths of the peptides in the antibody-peptide complex structures ranged from 5-20 residues, in accordance with the length cutoffs in selecting complexes for the set, yet these lengths were not evenly distributed, and many complexes had peptides of length between 8-11 residues (median length is 10 residues) (**Figure 1A**). While a subset of complexes contained peptides with Helix or Hairpin (or hairpin-like) classes, most antibody-bound peptides were classified as Coil or Other (**Figure 1B**). Likewise, the majority of peptide residues in the set were classified as having no regular secondary structure (irregular/loop, “C”; 54%), followed by turn (“T”; 13%) and α-helix (“H”, 13%) (**Figure 1C**). Representative structures of antibody-peptide complexes with three peptide structural classes (Helix, Hairpin, and Coil) are shown in **Figure 2**.

While the antibodies and therefore the antibody-peptide interfaces in the set of complexes are not redundant, we observed that some of the bound peptides had identical or overlapping sequences. To avoid possible resultant bias in peptide length or secondary structure, we grouped complexes with shared peptide sequences, selecting one complex per group based on resolution (“nr_epitope”, noted in **Table S1**). Analysis of lengths and secondary structures of this subset of the antibody-bound peptide structures is shown in **Figures 1D–F**); these are generally in accordance with the distribution of peptide lengths and secondary structure propensities of the full set of antibody-bound peptides. As with the full set of antibody-peptide complexes, the median length of the peptides in this subset is 10 residues, and comparison of the length values between the full and “nr_epitope” sets showed no statistically significant difference (p = 0.52, Wilcoxon rank-sum test).

### Interface Features and Comparison With Antibody-Protein Antigen Complexes

**Figure 3:**
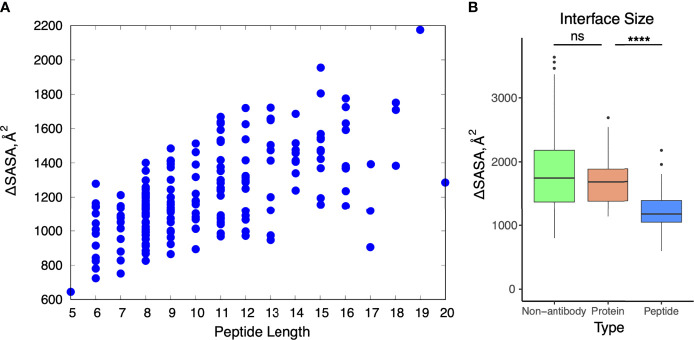
Buried solvent accessible surface area in antibody-peptide interfaces. **(A)** Change in accessible surface area (ΔSASA) for antibody-peptide interactions in comparison with length of the peptide (in residues) in the structure. **(B)** Change in accessible surface area for non-antibody-antigen (N = 190), antibody-protein (N = 54), and antibody-peptide (N = 188) interfaces. Non-antibody-antigen and antibody-protein complex structures are nonredundant sets previously reported in Protein Docking Benchmark 5.5 (7). Statistical significance tests between sets of values were calculated with Wilcoxon rank sum test (ns: p > 0.05; ****: p ≤ 0.0001). Two outlier points for the non-antibody-antigen set with very high ΔSASA values (6671 Å^2^, 6628 Å^2^) are not shown.

We focused our next analysis on interface features of the antibody-peptide complexes. First, we calculated binding interface sizes for the set (ΔSASA, **Figure 3**), reflecting the buried surface of both the antibody and peptide in the complex structure, as calculated previously for antibody-antigen interfaces (7). Interface buried surface areas ranged from approximately 600 Å^2^ to over 2000 Å^2^, and as expected, larger peptides were associated with larger buried interface surface areas (**Figure 3A**). We compared the binding interface sizes of these complexes with those of nonredundant non-antibody protein-protein complexes and antibody-protein antigen complexes from Docking Benchmark 5.5 (7). As only heavy-light chain antibodies are present in the antibody-peptide complexes in this study, single-chain nanobodies were excluded from the antibody-protein antigen set for this comparison. There was a pronounced observed difference in antibody-peptide binding interface sizes versus antibody-protein interface sizes (p ≤ 0.0001), whereas antibody-protein and non-antibody complex interfaces exhibited no significant difference in binding interface sizes (**Figure 3B**).

**Figure 4:**
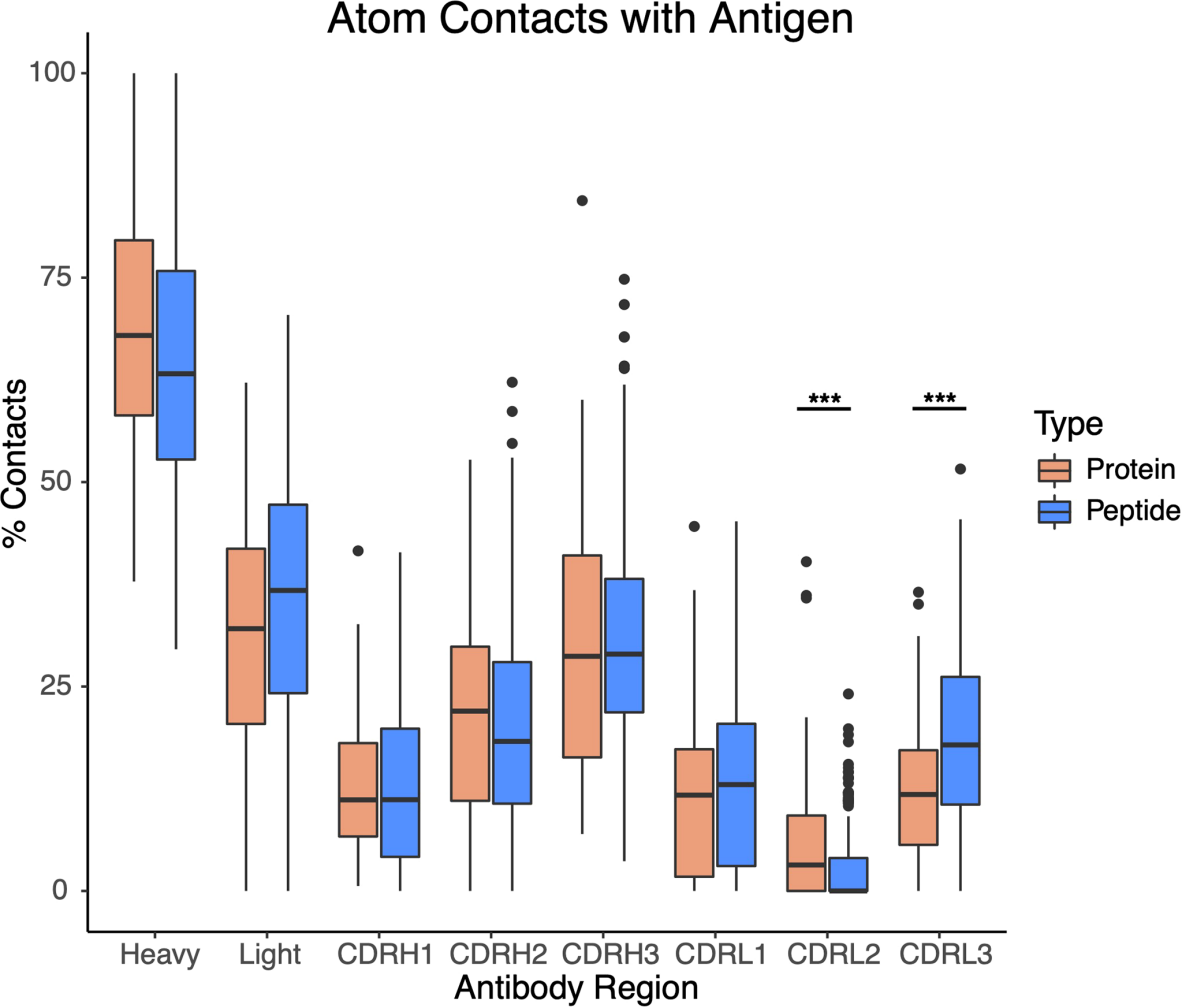
Contacts with antibody heavy chain and CDR loops for sets of antibody-protein (N = 54) and antibody-peptide (N = 188) interfaces. All atomic-level contacts between antigenic protein or peptide and the antibody (< 4.5 Å) were counted for each antibody-protein and antibody-peptide interface, and percentages of atomic contacts within each interface including the heavy chain (Heavy), light chain (Light), and the six heavy and light chain CDR loops were calculated. Statistically significant differences between sets of antibody-protein and antibody-peptide values (Wilcoxon rank sum test) are indicated (***: p ≤ 0.001).

Due to the difference in protein/peptide antigen sizes and interface buried surface areas, we examined the possibility that antibody-peptide interactions exhibited chain contact or CDR loop contact preferences that differed from those of antibody-protein interactions, due for instance to the peptide size preventing or limiting contacts with more peripheral and non-CDR3 loops, on average. However, we found that the preference for contacts with the antibody heavy chain was similar to that for the antibody-protein complexes, with many interfaces showing a moderate preference for the heavy chain (50-75% of antibody atomic contacts in most cases), and CDR loop contact preferences likewise showed no pronounced differences between the sets of antibody-protein and antibody-peptide complexes (**Figure 4**), with the exception of a significant decrease in percent of contacts involving CDRL2 for antibody-peptide complexes (p ≤ 0.001), and a significant increase in percent of contacts involving CDRL3 for antibody-peptide complexes (p ≤ 0.001). This decrease in relative atomic contacts by CDRL2 in antibody-peptide interfaces is likely due, at least in part, to the smaller size of peptide antigens leading to a lower likelihood of engaging the more peripheral CDRL2 loop on the antibody light chain, versus the CDRL3 loop which is more centrally located.

**Figure 5:**
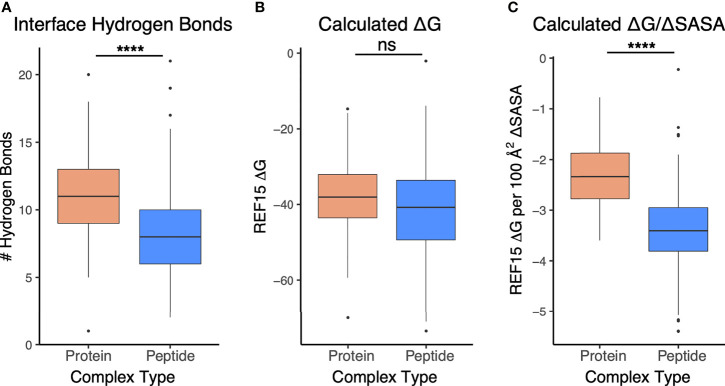
Interface and energetic features of antibody-peptide complexes in comparison with antibody-antigen complexes. **(A)** Interface hydrogen bonds, **(B)** Calculated binding affinity (ΔG), and **(C)** Calculated affinity per unit interface surface area are represented for antibody-antigen (N = 54) and antibody-peptide (N = 188) complex structures. Statistical significance between sets of values are from Wilcoxon rank sum test (ns: p > 0.05; ****: p ≤ 0.0001).

**Figure 6:**
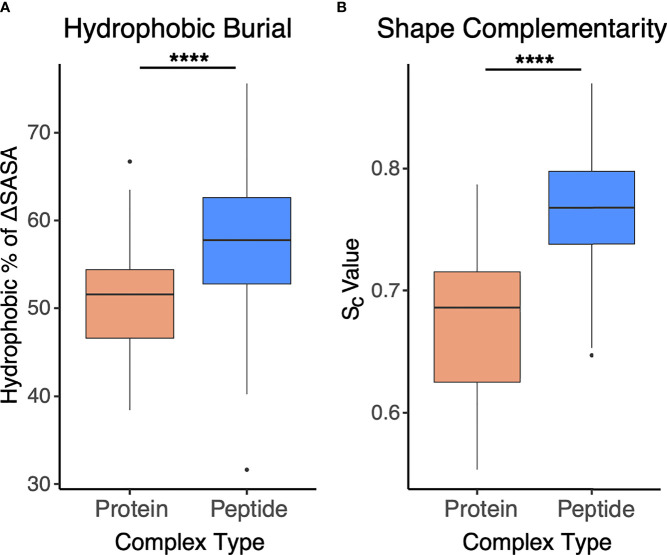
Interface hydrophobicity and shape complementarity of antibody-peptide versus antibody-protein complexes. **(A)** Percent of hydrophobic buried interface surface area (ΔSASA), calculated by Rosetta and **(B)** Shape complementarity values (Sc (38), calculated by Rosetta) from antibody-protein (N = 54) and antibody-peptide (N = 188) interface structures are represented. Statistical significance between sets of values are from Wilcoxon rank sum test (****: p ≤ 0.0001).

To assess and compare the sets of interfaces in more detail, particularly with regard to energetically relevant features, we calculated numbers of interface hydrogen bonds in the sets of interfaces, and we used Rosetta (44) to calculate binding affinities based on the complex structures (**Figure 5**). The numbers of hydrogen bonds were markedly lower in antibody-peptide interfaces, possibly due in part to the smaller interface sizes providing fewer opportunities to form such contacts (**Figure 5A**). However, Rosetta-predicted binding affinities, based on the REF15 potential which includes a range of energetic terms (37) and was recently found to be most accurate among a series of ΔG prediction functions for antibody-antigen affinity prediction (7), indicated that the antibody-peptide interface structures had binding energies similar to those of antibody-protein complexes (**Figure 5B**). Normalizing the calculated ΔG values by the interface buried surface area (ΔSASA) values to give energies per buried surface area showed there is significantly more energy density within the antibody-peptide interfaces (**Figure 5C**). To investigate possible reasons for this observation, we used Rosetta to calculate the fractional amount of hydrophobic atom burial and shape complementarity in antibody-peptide interfaces and antibody-protein interfaces (**Figure 6**). For both metrics, there was a clear and significant increase for antibody-peptide interfaces, indicating that a higher proportion of hydrophobic atom burial and improved shape complementarity allow antibody-peptide interactions to compensate for more limited interface sizes and achieve affinities comparable to antibody interactions with larger protein antigens. One caveat regarding the shape complementarity observation was previously described by Kuroda and Gray (25), who likewise found higher shape complementarities for a set of antibody-peptide interfaces versus antibody-protein interfaces, and noted that “edge effects” can potentially lead to high S_c_ values for peptides in particular.

**Figure 7:**
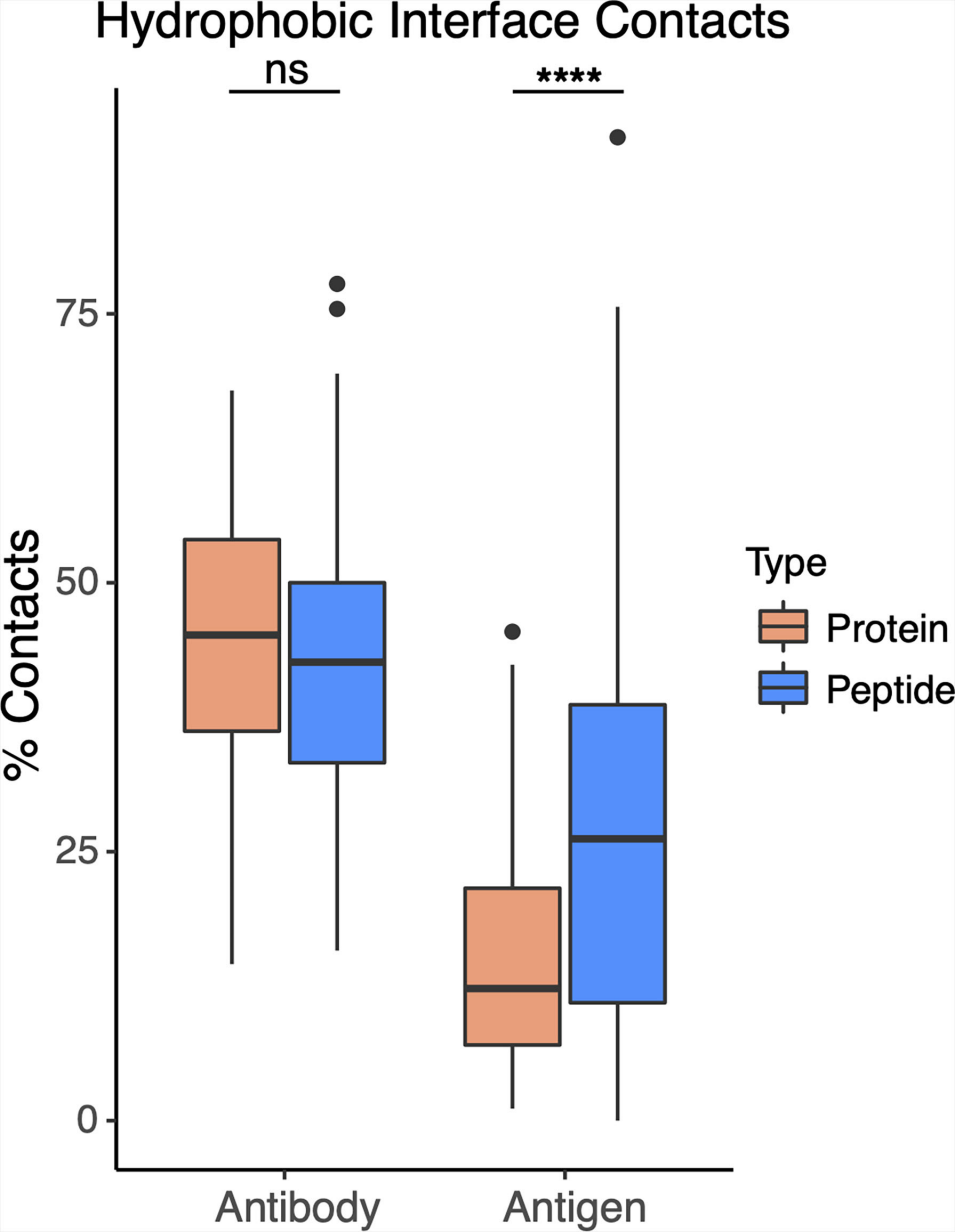
Hydrophobic atom contacts in antibody-peptide and antibody-protein complexes. Percent of total atom contacts (< 4.5 Å distance to binding partner) for hydrophobic residue side chains were calculated separately for antibody and antigen in each antibody-protein (N=54) and antibody-peptide (N=188) interface. Statistical significance between sets of values were determined by Wilcoxon rank sum test (ns: p > 0.05; ****: p ≤ 0.0001).

To assess whether higher hydrophobic atom composition in antibody-peptide interfaces is due to the antibody or antigen side of the interface, we calculated hydrophobic atom contacts as a total of all atom contacts for antibody and antigen sides of the interface, within the sets of antibody-protein and antibody-peptide interfaces (**Figure 7**). For these calculations, hydrophobic atoms were defined based on atom type classifications in the IFACE protein interface statistical potential (36) (detailed further in the Methods). While the antibody side of the interface did not show a change in hydrophobic atom contact percent, there was a significant increase (p ≤ 0.0001) in percent of hydrophobic atom contacts for the antigen side of the interface in antibody-peptide interfaces versus antibody-protein interfaces.

### Antibody Binding Conformational Changes

**Figure 8:**
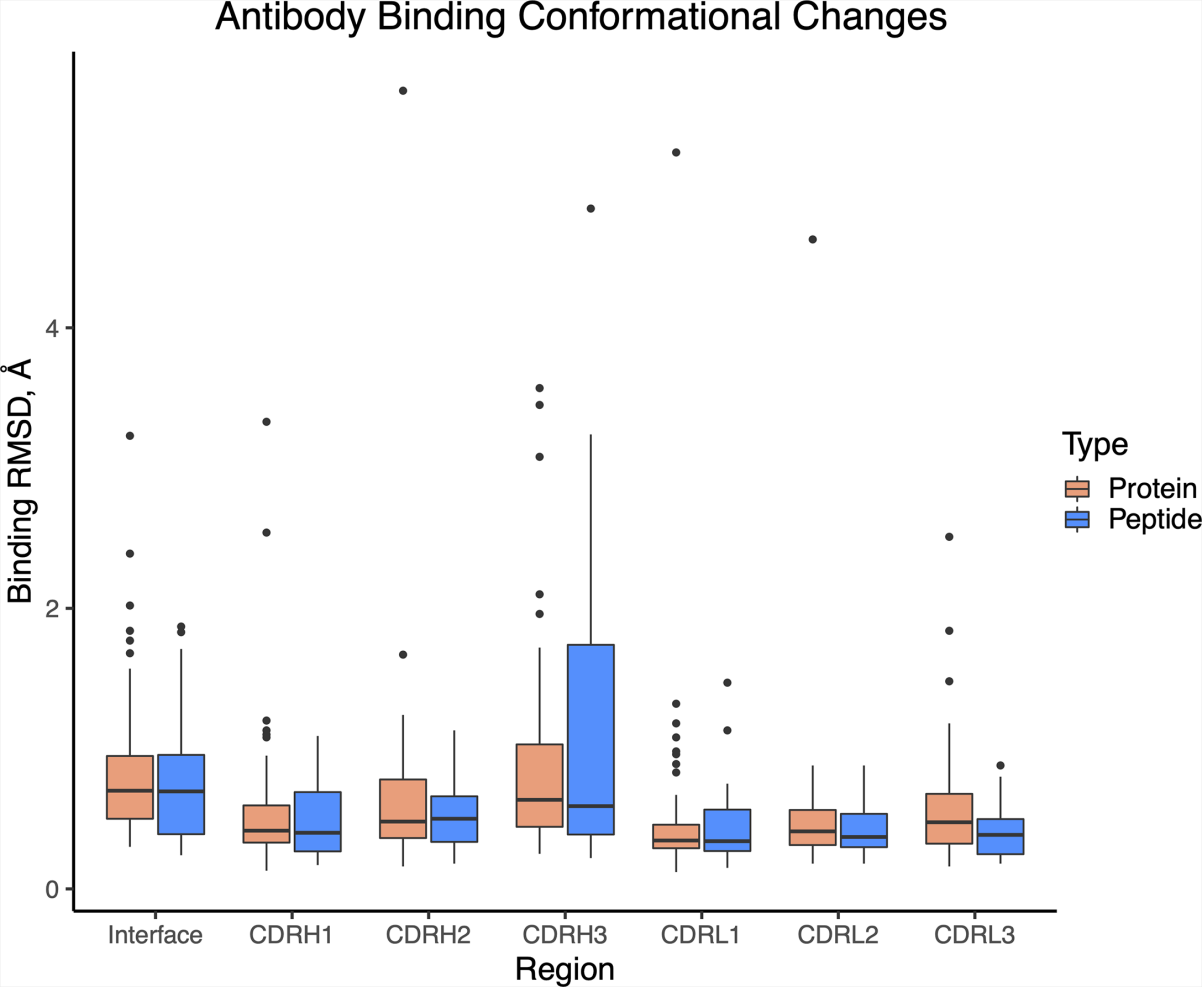
Antibody binding conformational changes in antibody-antigen and antibody-peptide complexes. Comparison of unbound and bound antibody structures was performed for antibody-peptide (N = 32) and antibody-antigen (N = 54) structures. Calculated values are backbone atom root-mean-square distances, using interface residues proximal (< 10 Å) of the bound antigen (Interface), or individual CDR loops.

Previously we found that antibody-antigen complexes exhibit various levels of binding conformational changes, ranging from relatively rigid “lock-and-key” recognition, to pronounced loop conformational changes (7). To examine whether such conformational changes are present in antibody-peptide complexes, we identified a set of 32 unbound antibody structures with antibodies matching corresponding antibody-peptide complexes in our set (**Table S2**), permitting us to calculate binding RMSD of peptide-proximal interface residues, as well as CDR loops (**Figure 8**). In spite of the smaller peptide sizes and interface surface areas, we found that levels of antibody binding conformational changes were similar between the antibody-peptide and antibody-protein complexes.

### Conformational Variability of Epitopes

**Figure 9:**
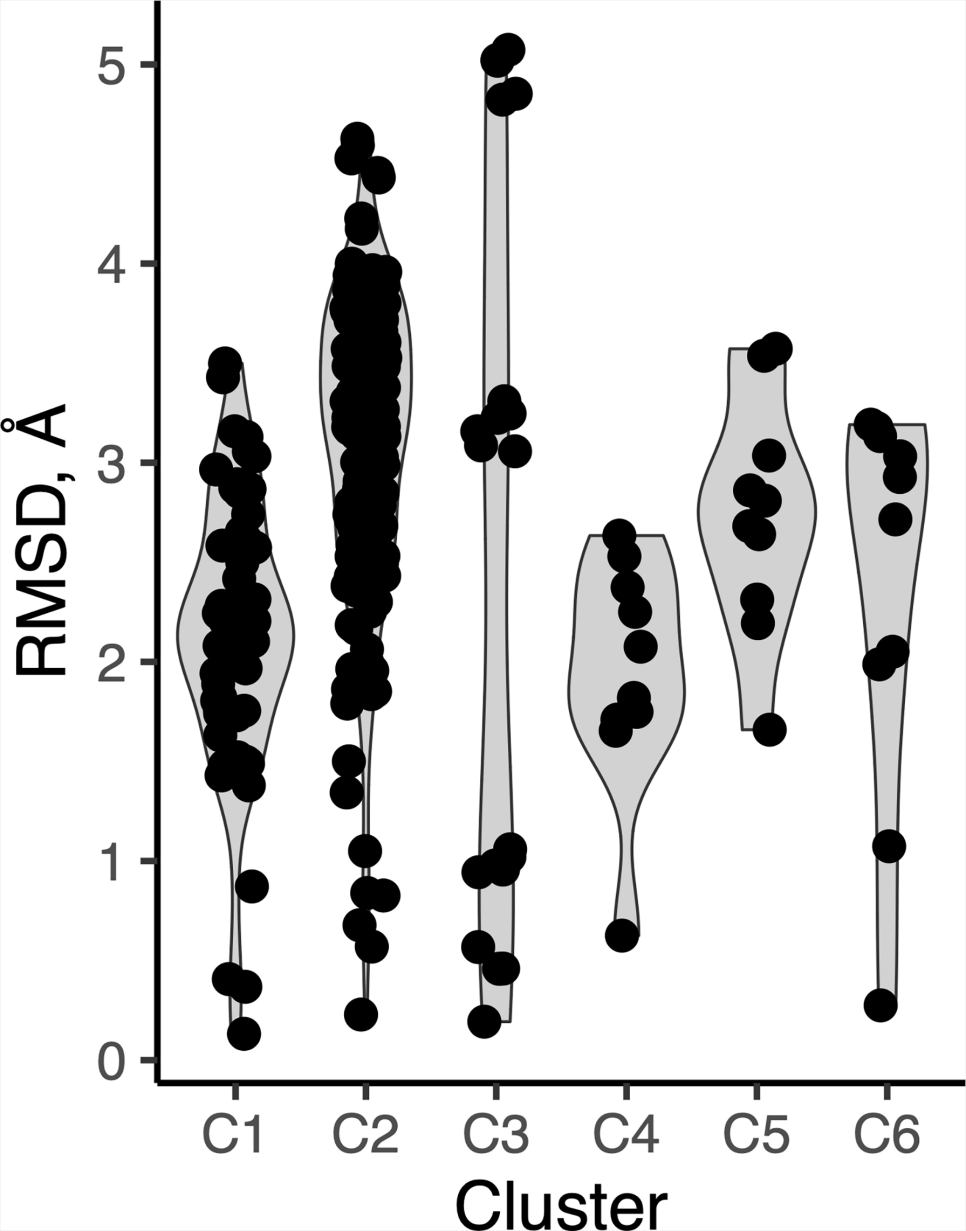
Peptide conformational variability in antibody-peptide complexes. Clusters of antibody-bound peptide structures (C1-C6), each with common epitope sequences, were used to calculate pairwise backbone root-mean-square distance between the observed peptide conformations within each set. Each point represents an RMSD value between two peptide conformations.

**Figure 10:**
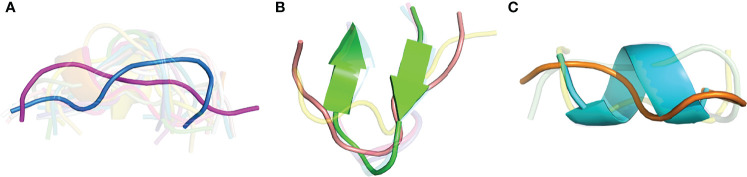
Structural variability of three peptide epitopes. Superposed structures of shared peptide residues from antibody-bound complexes for **(A)** Cluster 2, HIV fusion peptide (19 structures, 9 residues; AVGIGAVF), **(B)** Cluster 3, HCV E2 AS412 (7 structures, 9 residues; LINTNGSWH), and **(C)** Cluster 6, HIV Env MPER (5 structures, 6 residues; LELDKWA). For clarity, peptide structures are shown in semi-transparent cartoon representation, except for representative peptides from PDBs **(A)** 6PDU (blue) and 6NCP (magenta) (3.26 Å backbone RMSD), **(B)** 4DGY (green) and 4XVJ (salmon) (3.09 Å backbone RMSD), and **(C)** 1TJI (orange) and 5DD0 (cyan) (3.03 Å backbone RMSD).

As we observed several sets of antibody-peptide complexes with shared epitope sequences or subsequences, we compared the antibody-bound peptide conformations within those sets to assess levels of conformational variability or rigidity of those epitopes. Five sets (clusters) of complexes with shared epitopes were found with five members or more in each cluster (shown in **Table S3**). Those correspond to complexes with P. falciparum circumsporozoite protein (PfCSP) NANP repeat (cluster 1, or C1), HIV fusion peptide (C2), HCV E2 antigenic site 412 (AS412) (C3), PfCSP junctional epitope (C4), the HIV Env V3 loop (C5), and HIV Env membrane proximal external region (MPER) (C6). Backbone RMSD calculations were performed between common subsequences in all pairs of structures within each set (bold residues in **Table S3**), and all clusters were found to have a range of conformational variability (**Figure 9**). All clusters contained one or more pairs of epitope structures with at least moderate backbone RMSD (> 2 Å). Sets of peptide structures from Clusters 2, 3 and 6 are shown in **Figure 10**, and highlight the diversity of peptide structures within those clusters, including two pairs of peptides within each set with pronounced backbone conformational differences (~3 Å backbone RMSD).

## Discussion

This study provides a focused analysis of antibody-peptide complexes, highlighting features of that important class of antibody recognition. While we found that several properties of those interfaces are similar to those of antibody complexes with larger protein antigens, we also noted intriguing distinctions in calculated energy density, hydrophobicity, and shape complementarity that likely represent strategies used by antibodies to target smaller linear epitopes while retaining the capability to bind at high affinity *in vitro* and *in vivo*. We also observed considerable levels of peptide conformational variability within sets of structures with shared peptide epitopes; such conformational variability has been noted previously to represent a possible mechanism of viral evasion (45), while conversely it highlights the capacity of antibodies to effectively target a variety of distinct conformations.

Although in many cases the antibody-bound peptides represent the full epitopes of the corresponding antibodies, such as the HCV1 antibody which is known to bind a linear determinant in HCV E2 (12, 46), in some cases the native antibody affinity and activity may require the full antigen context, or indirect contributions of proximal residues or molecules. For instance, antibodies 10E8, 2F5, and 4E10, which engage the HIV Env MPER site, are known to be influenced by membrane lipids (47, 48). Relatedly, it should be noted that some antibodies represented in this analysis were obtained through vaccination with full antigen proteins or natural infection, while others were elicited by vaccination with designed or peptide antigens. While structurally resolved HETATM molecules were used to filter antibody-peptide interactions mediated in part by non-protein molecules from our analysis, it is possible that structurally unresolved molecules, including for instance N-glycans, could be a factor in peptide recognition for some antibodies. Prospective studies of these interfaces and structures could more explicitly account for experimentally measured antibody-peptide affinities, where available, analogous to the antibody-antigen affinities collected and analyzed as part of Protein Docking Benchmark 5.5 (7). Nevertheless, the high-quality set of interfaces considered here, and their core residue-level and atom level interactions, are likely reliable for comparative and/or dedicated analysis of antibody-peptide interface features.

Previously reported modeling approaches for protein-peptide interactions (28–30) and antibody-antigen interactions (26) have highlighted the importance of the consideration of peptide flexibility and antibody loop flexibility, respectively, thus antibody-peptide modeling represents a challenging area that requires the capability to sample peptide and antibody CDR loop backbone conformations. A recently described end-to-end deep learning approach (49) may be capable of effective sampling during the antibody-peptide modeling process, yet benchmarking has indicated that such approaches are not yet capable of reliably modeling antibody recognition (50). Development of comprehensive databases of antibody-peptide structures and identification of key interface features, as performed here, may enable future machine learning and data-driven approaches to accurately model and design such interactions, leading to development of next-generation immunotherapeutics and vaccines.

## Data Availability Statement

The original contributions presented in the study are included in the article/**Supplementary Material**. Further inquiries can be directed to the corresponding author.

## Author Contributions

Conceptualization: BP. Data curation: JL and BP. Funding acquisition: BP. Methodology: JL, RY, and BP. Visualization: JL and BP. Writing – Original Draft: BP. Writing – Review & Editing: JL, RY, GO, and BP. All authors contributed to the article and approved the submitted version.

## Funding

Funding for this work was provided by the National Institutes of Health (R01GM126299 and R35GM144083 to BGP).

## Conflict of Interest

The authors declare that the research was conducted in the absence of any commercial or financial relationships that could be construed as a potential conflict of interest.

## Publisher’s Note

All claims expressed in this article are solely those of the authors and do not necessarily represent those of their affiliated organizations, or those of the publisher, the editors and the reviewers. Any product that may be evaluated in this article, or claim that may be made by its manufacturer, is not guaranteed or endorsed by the publisher.
