# Supplementary material for: Structural Features of Antibody-Peptide Recognition
Source: Front Immunol. 2022 Jul 7;13:910367. doi: 10.3389/fimmu.2022.910367 (PMC9302003; doi:10.3389/fimmu.2022.910367)
Supplement: Supplementary file 1 [file DataSheet_1.pdf]

Table S1. Antibody-peptide complex structures.

PDB	Heavy chn	Light chn	Peptide chn	Resolution (Å)	PDB peptide sequence[1]	PDB peptide length[1]	ΔASA (A^2)[2]	# Interface Hbonds[3]	DSSP secondary structure[4]	Pep class[4]	NR epitope[5]
5xct	A	B	C	1.17	GYPGQV	6	780.6	7	CCCSCC	coil	1
4qyo	A	B	Q	1.21	NAYNMSIRR	9	1199.2	9	CHHHHSSCC	other	1
6wfy	H	L	P	1.23	NPNANPNANPNA	12	1297	10	CGGGCTTTCTTC	other	1
6ucf	H	L	A	1.29	AVGIGAVF	8	1096.2	8	CCCCCCCC	coil	1
7m53	H	L	A	1.4	SFKEELDKYF	10	1013.9	4	CHHHHHHHHC	helix	1
6vry	H	L	G	1.4	GLKRDKTKEYN	11	1352.7	12	CCCCCTTSCCC	coil	1
4tul	H	L	I	1.4	VCNPLTGALLC	11	1239.8	5	CCCTTTTTTTC	other	1
4xcf	H	L	P	1.43	NWWDITNWLWYIKKKK	16	1367.1	6	CGGGHHHHHHHHHHHC	helix	1
7doh	H	L	I	1.45	GTGATPADD	9	922.8	8	CCSSSGGGC	other	1
4hpy	H	L	P	1.5	KKQKVHALFYK	11	1638.8	11	CCCCTTGGGTC	other	1
3ifn	H	L	P	1.5	AEFRHD	6	1141	10	CCCCCC	coil	1
4hs6	H	L	Y	1.53	QLINTNGSWHIN	12	1065.8	8	CCEEETTEEECC	hairpin	1
5tkk	H	L	A	1.55	AVGIGAVF	8	1009.4	8	CCGGGCCC	coil	0
6pdr	H	L	A	1.56	AVGIGAVF	8	1219.7	4	CCSTTTTC	other	0
6pxr	H	L	A	1.56	AGTYGLGD	8	975	12	CCTTCCCC	coil	1
4yo0	C	D	F	1.56	VAMPGAEDDVV	11	1376.3	11	CCCTTCTTCCC	coil	1
6pbv	D	C	I	1.57	GNPDPNANP	9	1125.8	13	CCSSCCCCC	coil	1
3bkj	H	L	A	1.59	AEFRHDS	7	1212.4	8	CCCCCCC	coil	0
3g5y	B	A	E	1.59	CGADSYEMEEDGVRKC	16	1147.5	10	CCTTCCCEEETTEEEC	hairpin	1
6wg0	H	L	P	1.6	NPNANPNANPN	11	1241.1	13	CCCCSCSSCCC	coil	0
3uji	H	L	P	1.6	YNKRKRIHIGPGRAFYTT	18	1750.6	14	CCGGGSCEEETTTEECCC	hairpin	1
4n8c	H	L	X	1.6	SLLTEVETPIRNEWG	15	1194.6	10	CCCCTTTCSCGGGCC	other	1
4tqe	H	L	A	1.6	LPTPPTREPKKVAVVR	16	1725.1	12	CCCCCCSSCCCCCCCC	coil	1
6bzy	H	L	B	1.6	QLINTNGSWHI	11	1091	7	CCBCSSSCCBC	other	0
6azm	D	C	F	1.6	ANPNANPN	8	825.7	5	CCCCCCCC	coil	0
3v4u	H	L	P	1.64	EPQAPWME	8	976.1	3	CCSSTTTC	other	1
2v17	H	L	A	1.65	TDHGAE	6	723.8	6	CCCSCC	coil	1
2otu	D	C	Q	1.68	QQQQQQQQQQG	11	1668.5	15	CCCCCCCCSCC	coil	1
6vbo	H	L	C	1.68	KKQKVHALFYKLDIVP	16	1775.4	8	CHHHHHTTTTCCCCCC	other	0
5mo3	H	L	A	1.69	KHVPGGGS	8	911.7	9	CCCSSTTC	other	1
4hpo	H	L	P	1.69	DKKQKVHALFYKLDI	15	1804.5	14	CHHHHHHHHHCCCCC	helix	0
3ggw	B	A	E	1.7	YLEDWIKYNNQ	11	967.7	5	CHHHHHHHHHC	helix	1
3mly	I	M	Q	1.7	SIKIRPRQAFYA	12	1415.2	11	CCCCCTTCCCCC	coil	1

6mtt	H	L	P	1.7	KWASLWNW FDITKWLWYI K	19	2175.4	14	CCCCCCTT SCTTTHHH HHC	other	1
6x7w	A	C	D	1.7	AVGLGAVF	8	1135.9	7	CCCCCCCC	coil	1
5nph	H	L	A	1.7	NETDVMLLN	9	1170.8	4	CTTHHHHT C	other	1
6pec	H	L	A	1.75	AVGIGAVF	8	1151.5	3	CCGGGGCC	other	0
7rd4	H	L	G	1.75	NPDPNANPN VDPNAN	15	1538.2	13	CCCTTSSTT CCTTCC	other	0
5u3o	H	L	A	1.76	KWNWFDITN WLWYIRKK	17	1392.2	6	CCCHHHHH HHHHHHHT C	helix	1
6bqb	H	L	P	1.77	NPDPNAN	7	879.8	4	CCCCSCC	coil	0
6b5r	H	L	A	1.78	NPDPNANPN VDPN	13	1200	8	CCCTTSCS CSCCC	coil	0
1mvu	B	A	P	1.78	VVQEALDKA REGR	13	974	6	CHHHHHHH HHHTC	helix	1
6vjt	H	L	P	1.78	STKPSDGNS T	10	893.5	7	CBCTTTCC BC	other	1
6p7h	A	B	C	1.78	AVGIGAVF	8	1149.6	8	CCCCGGGC	coil	0
4dgy	H	L	A	1.8	QLINTNGSW HIN	12	971	6	CEEEETTEE EEC	hairpin	0
1pz5	B	A	C	1.8	MDWNMHAA	8	1163.2	5	CCCSTTTC	other	1
1i8k	B	A	C	1.8	KKGNYVVTD H	10	1158.4	14	CBTTBCSS CC	other	1
3fn0	H	L	P	1.8	WNWFDITNK	9	1001	8	CCSSCTTC C	other	1
6uoe	H	L	P	1.8	NETIYNTTLK YG	12	1537.2	12	CCCCCCCC CCCC	coil	1
3mlr	H	L	P	1.8	TKKGIAIGPG RTLY	14	1414	8	CTTCEEEET TEEEC	hairpin	1
3mnz	B	A	P	1.8	NAQELLELD KWASLWN	16	1234.9	6	CHHHHHHH HHHHTTCC	helix	1
6mnq	H	L	P	1.8	KSIRIGPGQA FY	12	997.6	5	CCEEEETTE EEC	hairpin	1
6wfz	H	L	P	1.84	NPNANPNAN PN	11	985.5	7	CGGGCTTS CCC	other	0
6d0x	A	B	C	1.85	NANPNANPN AN	11	1077.5	7	CCCTTSCC SCC	coil	0
4o4y	H	L	A	1.85	PAPELLG	7	952.9	10	CCCCCCC	coil	1
6w00	H	L	P	1.85	NPNANPNA	8	1195.2	8	CTTSCCCC	coil	0
1svz	A	A	C	1.89	PQFSLW	6	1148.4	9	CCCCCC	coil	1
3go1	H	L	P	1.89	RKRIHIGPGR AFYT	14	1406.3	14	CCCCCCCT TCCCCC	coil	0
6pds	B	A	C	1.89	AVGIGAVF	8	1047	4	CCGGGCCC	coil	0
7s3n	H	L	A	1.9	SFKEELDKYF	10	1102.8	3	CHHHHHHH HC	helix	0
6lra	H	L	C	1.9	VQIINK	6	914.3	10	CCCCCC	coil	1
1ce1	H	L	P	1.9	GTSSPSAD	8	867.4	13	CCCTTCC	coil	1
1e4x	I	M	Q	1.9	VVSHFND	7	1014.7	8	CCSSTTC	other	1
6zvf	H	L	P	1.9	QAPPGAYPG	9	1073.1	5	CCCGGGSC C	other	1
5v6m	H	L	P	1.9	TRKSIHIGPG RAF	13	1657.6	10	CCCSCCCS SSCCC	coil	1
6ct7	A	B	T	1.9	MDVFMKGLS K	10	1512.8	7	CCCSCCCC CC	coil	1
6ubi	D	E	F	1.9	AVGIGAVF	8	1126.5	3	CCCSSCCC	coil	0
4lkx	A	B	R	1.92	LAGGSAQSQ RAPDR	14	1515	10	CTTSGGGS SSSCCC	other	1

6o28	C	D	F	1.93	GNPDPNANPN	10	1011.9	5	CCCCTTSCCC	coil	0
5gir	H	L	C	1.93	KPIIIGSHAYGD	12	1324	13	CCCCTTHHHHCC	other	1
2brr	H	L	P	1.95	VVNNKVATH	9	864.4	6	CEETTEECC	hairpin	1
5eoq	H	L	A	1.95	REAIVKADE	9	1244.8	14	CCCCCCCCC	coil	1
3o41	A	B	C	1.95	NRGIIKTFS	9	1200.3	9	CCCCCSCCC	coil	1
1qkz	H	L	P	1.95	ANGGASGQVK	10	1081.4	5	CCSBTTBCCC	other	1
6pdu	H	L	C	1.95	AVGIGAVF	8	1226.3	7	CCSSCCCC	coil	0
5t6p	B	A	F	1.97	DTRPAP	6	1101.6	6	CCCCCC	coil	1
3lex	A	B	C	1.97	LELDKWA	7	1076.3	9	CCCCTTC	coil	0
5eoc	J	M	Q	1.98	CQLINTNGSWHIC	13	947.3	4	CEEEESSSEEEEC	hairpin	0
6mqc	H	L	C	1.99	AVGIGAVF	8	1354.5	10	CCSCCCCC	coil	0
4ojf	H	L	A	2	DAEFRH	6	1044	9	CGGGTC	other	1
5ea0	H	L	P	2	PPPIDNGDITS	11	1254.4	6	CCCCCGGGTCC	coil	1
4h0h	B	B	D	2	FYPYPYA	7	1134.6	2	CCSCCCC	coil	1
2b1h	H	L	P	2	TKKSIKIRPRQAFYAT	16	1592	10	CCSCBCSSTTCCBSCC	other	0
4onf	H	L	P	2	DAEFRH	6	1008.5	9	CGGGTC	other	0
3ffd	A	B	P	2	SIQDLRRRFFLHHLIAEI	18	1708.7	21	CHHHHHHHHHHHHHHHCC	helix	1
4z0x	B	A	C	2	TGWLAGLFYQHK	12	1313.8	6	CHHHHHHHCCCC	helix	1
6dcw	H	L	T	2	YKPVDLSKV	9	1158	6	CCCCCCCCC	coil	1
4xvj	H	L	A	2	RQLINTNGSWHIN	13	1721.7	10	CCCCCCSSCSCCC	coil	0
2qhr	H	L	P	2	VEQHHRRTDND	11	1630	19	CCCCCCCCTTC	coil	1
7lki	A	B	C	2	GAPTYSWG	8	1245.8	11	CCCSSSCC	coil	1
7jwq	C	D	P	2	LFFEVD	6	983.4	11	CCCSCC	coil	1
6pef	D	E	F	2	AVGIGAVF	8	1259.1	7	CTTCCSCC	coil	0
2hkf	H	L	P	2.01	LPGEEDLPG	9	1301.7	11	CCCSCCSCC	coil	1
4jo1	I	M	Q	2.03	TRKSIHIGPG	10	1317.9	10	CCCSCCCSCC	coil	0
6mtp	A	B	Q	2.04	WNWFDITKWLWYIKK	15	1474.8	3	CCGGGHHHHHHHHHC	helix	1
5aum	A	B	D	2.05	RENLYFQGKDG	11	1592.3	17	CHHHHHHCCCC	helix	1
6pbw	D	C	E	2.06	NPNANPNA	8	1088	12	CCCCCCCC	coil	0
5zv3	H	L	A	2.09	EEPGSETSDAKS	12	1090.2	15	CCCGGGTTTCCC	other	1
6cdo	A	B	C	2.1	AVGIGAVF	8	992	7	CCCTTTTC	other	0
3o6l	H	L	C	2.1	EPVDPKLEPWKHPGS	15	1423.1	10	CCSSCCCCTTCCCCC	coil	1
1a3r	H	L	P	2.1	VKAETRLNPDLQPTE	15	1955	19	CCCCTTSCGGGCSCC	other	1
3e8u	H	L	P	2.1	GVQGSGAFGR	10	1179.5	9	CCSCGGGGC	other	1
6p8d	A	B	C	2.1	AVGIGAVF	8	1298.3	6	CCCSSSCC	coil	0

6axk	A	B	E	2.1	NPNANPNANPN	11	1208.8	8	CTTSCCSSSCC	other	0
6vbq	C	D	K	2.12	KKQKVHALFYKLD	13	1648.5	9	CCCSSCHHHHHHC	other	0
5tkj	G	H	I	2.12	AVGIGAVF	8	996.1	9	CCCTTCCC	coil	0
7k76	A	B	P	2.14	LRKPKHKKLKQ	11	984.1	10	CCSCCCCCCCC	coil	1
3ifo	A	B	Q	2.15	AEFRHD	6	1164.6	15	CCCCCC	coil	0
7nab	A	B	D	2.15	DSFKEELDKYFKNHTSPDVD	20	1285.2	12	CHHHHHHHHHHTSSCCTTCC	other	0
5tbd	D	G	H	2.2	NKENCGAA	8	1024.2	12	CTTTTSCC	other	1
2hrp	H	L	P	2.2	MSLPGRWKPK	10	1174.8	8	CCBTTTBCCC	other	1
5xcs	A	B	C	2.2	YPYDVPDYA	9	1139.4	12	CCSCCCCCC	coil	1
5dlm	H	L	X	2.2	SLLTEVETP	9	1410.3	9	CTTTSSSCC	other	0
1uwx	M	K	Q	2.2	HFVQQTPKSQPTL	13	1121.7	2	CCBCCSTTSCCBC	other	1
1tji	H	L	P	2.2	EQELLELDKWASLW	14	1475.7	13	CCSCCCCCTTTTCC	coil	0
6kva	H	L	B	2.2	FEDFWK	6	1278.3	5	CCCTTC	coil	1
2ck0	H	L	P	2.2	CKEWLSTAPCG	11	1039.1	6	CCSTTCCSCCC	coil	1
6mnr	H	L	P	2.2	KSIRIGPGQAFYAT	14	1339.3	4	CCEEETTTTEEEEC	hairpin	0
4nrx	H	L	P	2.21	NEQELLELDKWASL	14	1685.8	10	CTTGGGGGCCSSCC	other	0
6db7	I	M	Q	2.21	RKRIHIGPGRAFY	13	1657.4	6	CCCEEEEETTEEEC	hairpin	0
4wht	S	T	s	2.22	QLINTNGSWHV	11	1532.7	10	CCSCTTCTTCC	other	0
5eor	H	L	A	2.27	VQTGRRPYE	9	1414.3	12	CCCCCCSCC	coil	1
6vbp	C	D	E	2.3	VHALFYKLDIVPI	13	1504.1	8	CCCTTCCTTCCCC	coil	1
4zto	H	L	P	2.3	GKAMYAPPIRG	11	1282.8	8	CCCCCCCCCCC	coil	1
2g5b	D	C	J	2.3	PTSSEQI	7	1090	12	CGGGCCC	other	1
3eyf	B	A	E	2.3	TIYNTTLKY	9	1408.3	9	CCCCCCCCC	coil	0
1ejo	H	L	P	2.3	YTTSTRGDLAHVT	13	1413.1	11	CCSCSSSTTSSCC	other	1
6n16	A	B	E	2.3	AVGIGAVF	8	1219.3	7	CCCCSTTC	coil	0
4j8r	D	C	J	2.3	PHGGSWGQ	8	1139	4	CCSCCCCC	coil	1
5vzy	H	L	A	2.32	HHQKLVFFAEDV	12	1719.9	7	CCCCCCCSTTCC	coil	1
1n64	H	L	P	2.34	PGGGQIVGGVYLLPRR	16	1370.2	8	CTTCCCCCGGGCCCCC	coil	1
1p4b	H	L	P	2.35	AHLENEVARLKK	12	1249.4	3	CHHHHHHHHHTC	helix	1
6x78	H	L	G	2.36	AVGLG	5	644.4	4	CTTCC	coil	0
4yr6	D	E	F	2.38	KLRGVLQGHL	10	1220.5	13	CCCCGGGTCC	coil	1
6cez	H	L	P	2.4	RDKVQKEYALFYKL	14	1238.5	5	CCHHHHHHHHHHTC	helix	1
6axl	A	B	I	2.4	NPNANPNANPNA	12	1286.8	7	CTTCCGGGCTTC	other	0
3hr5	H	L	R	2.4	AQSQRAPDR	9	1373.9	16	CCCCCSCCC	coil	0

5iq9	A	B	C	2.4	ASLWNWFDITNWLWYIRR	18	1383.1	2	CTTSCHHHHHHHHHHHHC	helix	1
2h1p	H	L	P	2.4	LQYTPSWMLVG	11	1252.2	7	CCCCCTTTSCC	coil	1
3ghe	H	L	P	2.4	RKRIHIGPGRAFYAT	15	1568.5	12	CCEEEEETTEEEEEC	hairpin	0
4bh8	B	A	P	2.4	RPSYISHL	8	923.3	5	CCCCCSCC	coil	1
6mqr	H	L	A	2.45	AVGIGAVF	8	1052.6	4	CCGGGCCC	coil	0
6cdp	A	B	C	2.46	AVGIGAVF	8	1202	9	CCCSSSCC	coil	0
6mqe	A	B	D	2.46	AVGIGAVF	8	1400.4	11	CCSCCCCC	coil	0
5dd0	H	L	P	2.49	LLELDKWASLW	11	1070.6	3	CHHHHHHHHHC	helix	0
6lz4	D	E	F	2.49	GDGMVPP	7	1078.1	5	CCTTSCC	other	1
6p60	A	B	E	2.5	AVGIGAVF	8	1208.4	3	CCSSCCCC	coil	0
5ijk	A	C	X	2.5	LQPEQPFP	8	1049.9	4	CCCSSCCC	coil	1
3u0t	B	A	F	2.5	AIIGLMVGGVV	11	1300.3	8	CCTTSSTTCCC	other	1
4rav	C	D	F	2.5	TLEKLMKAFESLKSF	15	1155.5	7	CHHHHHHHTCCCCCC	other	1
1kcs	H	L	P	2.5	QLDPAFG	7	827.3	4	CCCGGGC	other	1
1kc5	H	L	P	2.5	HQLDPAFG	8	952	6	CCCCTTCC	coil	0
6w05	H	L	P	2.52	NPNANPNA	8	1178.9	9	CTTSCCCC	coil	0
5drz	B	A	Q	2.54	IWGCSGKLICTTAVP	15	1545.2	13	CCCCSSSTTTTTSCC	other	1
5v6l	I	M	Q	2.55	HIGPGRAFYTTGEIIG	16	1381.6	7	CCCTTHHHHHHCCCCC	other	1
1hi6	B	A	C	2.55	DATPEWLGARL	11	1416.7	7	CCCSTTCCSCC	coil	1
7o2z	H	L	P	2.55	APAPAAPA	8	884.7	3	CCCCSSCC	coil	1
6ule	C	D	I	2.55	NPNANPNANPNANPNAN	17	1118.4	8	CTTSCCSCCTTSCCSCC	other	0
1nak	H	L	P	2.57	KRIHIGPGRA	10	1067.7	6	CCCCCSSCCC	coil	0
6wfx	H	L	P	2.59	NPNANP	6	824.6	8	CCSCCC	coil	0
6db5	I	M	Q	2.6	KKGIAIGPGRTLYA	14	1457.4	5	CCCEEEETTEEECC	hairpin	1
2eh8	H	L	P	2.6	ANSNNPDWDF	10	1390.1	11	CCSSSGGGCC	other	1
6fy3	X	Y	Z	2.6	LRDKKQKAYALFYRPD	16	1630.3	8	CCCHHHHHHHHHCCCC	helix	1
6o42	B	A	I	2.6	NWFDITNWLWYIKKK	15	1368.9	6	CHHHHHHHHHHHHHC	helix	1
1f90	H	L	E	2.6	KPLEEVLNL	9	994.1	4	CCHHHHTTC	other	1
4jo3	H	L	P	2.6	EIIGDIRQA	9	1484	13	CCCSCGGGC	other	1
6f7t	B	A	D	2.6	ERRNELQKQENR	12	1625.4	15	CCCCTTCCTTCC	coil	1
4hs8	H	L	A	2.6	QLINTNGSWHIN	12	1118.9	6	CEECSSSSCEEC	hairpin	0
3bky	H	L	P	2.61	CEPANPSEKNSPSTQYC	17	905.2	2	CCCSSTTSCSSSSTTTC	other	1
6bzu	C	D	I	2.7	LINTNGSWH	9	961.9	6	CCEETTEEC	hairpin	0
5wtt	H	L	P	2.7	CGLECNFG	8	1088.5	3	CTHHHHHC	helix	1
4ydv	B	A	Q	2.7	WGCSGKLICTT	11	1245	11	CCCSSSHHHHC	other	0

2or9	H	L	P	2.7	EQKLISEEDLN	11	1522.2	10	CCCCCCSTTTC	coil	1
1xgy	I	M	Q	2.71	TGALQE	6	843.4	7	CHHHHC	helix	1
6ncp	C	D	E	2.76	AVGIGAVFL	9	1050.8	8	CCCCCCCCC	coil	0
1frg	H	L	P	2.8	DVPDYASL	8	1106.3	10	CCCCTTCC	coil	1
6wn4	A	B	C	2.8	DSYFSWSDWWS	11	1075.3	10	CCCCCHHHHHC	other	1
3dsf	H	L	P	2.8	LNPDPSQ	7	751.4	9	CCSSCCC	coil	1
2igf	H	L	P	2.8	EVVPHKK	7	1148.3	12	CCCTTCC	coil	1
1ggi	H	L	P	2.8	CKRIHIGPG	9	1118.9	5	CCCCCCSCC	coil	1
2y6s	D	C	P	2.8	KLGLITNTIAGVAGL	15	1466.7	5	CCCCCCSSCSSCSCC	coil	1
2gsi	H	G	Z	2.81	PKKGVEKY	8	1125	9	CCTTTCCC	coil	1
4hzl	A	B	F	2.85	NESLNTGWLAGLF	13	1473.9	9	CCCSCCSGGGGGC	other	1
2hh0	H	L	P	2.85	HGQWNKPSK	9	1392.8	5	CCSSSCSCC	other	1
4q0x	H	L	E	2.9	NTGWLAGLF	9	1109.4	2	CCCHHHHHC	helix	0
1cu4	H	L	P	2.9	APKTNMKHMA	10	1462.7	8	CCCSSCCCC	coil	1
6mqs	A	B	E	3	AVGIGAV	7	1051.2	2	CCSSCCC	coil	0
1fpt	H	L	P	3	DNPASTTNKDK	11	1052	7	CCCCTTSCTTC	other	1

[1]Peptide sequence and peptide length, representing residues present in the PDB structure of the antibody-peptide complex (unresolved terminal peptide residues, if any, not included).
[2]Change in solvent accessible surface area value for the antibody-peptide complex, calculated using NACCESS (1).
[3]Number of antibody-peptide interface hydrogen bonds, calculating using hbplus (2).
[4]Peptide residue secondary structure classifications, calculated by DSSP (3), and peptide-level structure classifications.
[5]Antibody-peptide complex present in the nonredundant epitope sequence (“nr_epitope”) subset of complexes (N = 121).

References

1. Hubbard SJ, Thornton JM. *Naccess*. 2.1.1 ed: Department of Biochemistry and Molecular Biology, University College London (1993).
2. McDonald IK, Thornton JM. Satisfying Hydrogen Bonding Potential in Proteins. *Journal of molecular biology* (1994) 238(5):777-93. Epub 1994/05/20. doi: 10.1006/jmbi.1994.1334.
3. Kabsch W, Sander C. Dictionary of Protein Secondary Structure: Pattern Recognition of Hydrogen-Bonded and Geometrical Features. *Biopolymers* (1983) 22(12):2577-637. doi: 10.1002/bip.360221211.
4. Lawrence MC, Colman PM. Shape Complementarity at Protein/Protein Interfaces. *Journal of molecular biology* (1993) 234(4):946-50. Epub 1993/12/20. doi: 10.1006/jmbi.1993.1648.
5. Leman JK, Weitzner BD, Lewis SM, Adolf-Bryfogle J, Alam N, Alford RF, et al. Macromolecular Modeling and Design in Rosetta: Recent Methods and Frameworks. *Nat Methods* (2020) 17(7):665-80. Epub 2020/06/03. doi: 10.1038/s41592-020-0848-2.
