# Supplementary material for: Structural Features of Antibody-Peptide Recognition
Source: Front Immunol. 2022 Jul 7;13:910367. doi: 10.3389/fimmu.2022.910367 (PMC9302003; doi:10.3389/fimmu.2022.910367)
Supplement: Supplementary file 2 [file DataSheet_2.pdf]

Table S2. Complex and unbound antibody structures, and binding root mean square distance (RMSD) values.

Complex PDB	Unbound PDB	Antibody interface RMSD (Å)	CDRH1 RMSD (Å)	CDRH2 RMSD (Å)	CDRH3 RMSD (Å)	CDRL1 RMSD (Å)	CDRL2 RMSD (Å)	CDRL3 RMSD (Å)
4tul	4tuo	0.94	1.09	1	0.38	0.56	0.3	0.52
4xcf	4xcn	0.46	0.58	0.47	0.73	0.22	0.38	0.35
4yo0	4yny	0.43	0.35	1.13	0.46	0.21	0.19	0.57
3bkj	3bkm	0.33	0.32	0.32	0.45	0.28	0.19	0.24
4tqe	4tpr	1.58	0.54	0.65	2.88	0.33	0.53	0.36
2v17	3l1o	0.29	0.3	1.13	0.51	0.27	0.36	0.22
5mo3	5mvj	0.39	0.33	0.36	0.46	0.35	0.88	0.27
3ggw	3c6s	0.24	0.27	0.32	0.25	0.33	0.67	0.21
1pz5	1m7i	0.33	0.26	0.29	0.34	0.27	0.3	0.31
3mnz	3mo1	0.97	0.79	0.54	0.69	0.46	0.55	0.49
4o4y	4ma3	0.95	0.81	0.83	0.7	0.39	0.33	0.7
4h0h	5vf2	0.3	0.17	0.21	0.26	0.33	0.18	0.18
6dcw	6dcv	0.39	0.24	0.34	0.34	0.4	0.3	0.18
3o6l	3o6k	0.44	0.24	0.28	0.4	0.71	0.33	0.21
1a3r	1bbd	0.97	0.84	1.02	1.77	0.45	0.48	0.34
2hrp	1mf2	0.8	0.32	0.65	0.99	1.13	0.62	0.39
2ck0	1ck0	0.27	0.19	0.18	0.31	0.24	0.21	0.2
4zto	4ztp	0.49	0.66	0.57	0.29	0.15	0.29	0.46
5vzy	5vzx	0.68	0.48	0.49	0.59	0.25	0.27	0.45
1n64	1nlb	0.41	0.22	0.36	0.22	0.64	0.53	0.38
1p4b	1p4i	0.84	0.59	0.69	1.73	0.32	0.48	0.8
5ijk	5ik3	1.87	0.78	0.49	3.24	0.59	0.24	0.8
1kcs	1kcv	1.02	0.33	0.41	1.97	0.29	0.34	0.88
1kc5	1kcu	0.38	0.25	0.28	0.59	0.22	0.23	0.39
1hi6	1cfq	0.72	0.6	0.51	0.39	0.37	0.72	0.42
2eh8	2eh7	1.83	0.81	0.77	4.85	0.6	0.64	0.49
1f90	1f8t	0.48	0.24	0.22	0.54	0.58	0.3	0.25
4jo3	4jo4	1.21	0.37	0.43	1.89	0.22	0.44	0.23
6wn4	6wt3	0.71	0.43	0.85	0.78	0.28	0.77	0.48
2igf	1igf	0.9	0.58	0.55	0.88	1.47	0.63	0.58
1ggi	1ggc	1.71	0.94	0.57	2.16	0.75	0.42	0.56
1cu4	1cr9	0.86	0.91	0.62	2.02	0.42	0.38	0.25
