# Supplementary material for: Structural Features of Antibody-Peptide Recognition
Source: Front Immunol. 2022 Jul 7;13:910367. doi: 10.3389/fimmu.2022.910367 (PMC9302003; doi:10.3389/fimmu.2022.910367)
Supplement: Supplementary file 3 [file DataSheet_3.pdf]

Table S3. Clusters of complexes, with ≥ 5 members each, based on shared peptide sequences.

PDB	Resolution	PDB sequence[1]	DSSP[2]	Peptide class[2]
**Cluster 1**				
6wfy	1.23	NPNA**NPNANP**NA	CGGGCTTTCTTC	other
6wg0	1.6	NPNA**NPNANP**N	CCCCSCSSCCC	coil
6azm	1.6	A**NPNANP**N	CCCCCCCC	coil
6wfz	1.84	NPNA**NPNANP**N	CGGGCTTSCCC	other
6d0x	1.85	NA**NPNANP**NAN	CCCTTSCCSCC	coil
6w00	1.85	**NPNANP**NA	CTTSCCCC	coil
6pbw	2.06	**NPNANP**NA	CCCCCCCC	coil
6axk	2.1	NPNA**NPNANP**N	CTTSCCSSSCC	other
6axl	2.4	NPNA**NPNANP**NA	CTTCCGGGCTTC	other
6w05	2.52	**NPNANP**NA	CTTSCCCC	coil
6ule	2.55	NPNA**NPNANP**NANPNAN	CTTSCCSCCTTSCCSCC	other
6wfx	2.59	**NPNANP**	CCSCCC	coil
**Cluster 2**				
6ucf	1.29	**AVGIGAVF**	CCCCCCCC	coil
5tkk	1.55	**AVGIGAVF**	CCGGGCCC	coil
6pdr	1.56	**AVGIGAVF**	CCSTTTTC	other
6pec	1.75	**AVGIGAVF**	CCGGGGCC	other
6p7h	1.78	**AVGIGAVF**	CCCCGGGC	coil
6pds	1.89	**AVGIGAVF**	CCGGGCCC	coil
6ubi	1.9	**AVGIGAVF**	CCCSSCCC	coil
6pdu	1.95	**AVGIGAVF**	CCSSCCCC	coil
6mqc	1.99	**AVGIGAVF**	CCSCCCCC	coil
6pef	2	**AVGIGAVF**	CTTCCSCC	coil
6cdo	2.1	**AVGIGAVF**	CCCTTTTC	other
6p8d	2.1	**AVGIGAVF**	CCCSSSCC	coil
5tkj	2.12	**AVGIGAVF**	CCCTTCCC	coil
6n16	2.3	**AVGIGAVF**	CCCCSTTC	coil
6mqr	2.45	**AVGIGAVF**	CCGGGCCC	coil
6cdp	2.46	**AVGIGAVF**	CCCSSSCC	coil
6mqe	2.46	**AVGIGAVF**	CCSCCCCC	coil
6p60	2.5	**AVGIGAVF**	CCSSCCCC	coil
6ncp	2.76	**AVGIGAVF**L	CCCCCCCCC	coil
6mqs	3	AVGIGAV	CCSSCCC	coil
**Cluster 3**				
4hs6	1.53	Q**LINTNGSWH**IN	CCEEETTEEECC	hairpin
6bzy	1.6	Q**LINTNGSWH**I	CCBCSSSCCBC	other
4dgy	1.8	Q**LINTNGSWH**IN	CEEEETTEEEEC	hairpin

5eoc	1.98	CQLINTNGSWHIC	CEEEESSSEEEEC	hairpin
4xvj	2	RQ**LINTNGSWHI**N	CCCCCCSSCSCCC	coil
4wht	2.22	Q**LINTNGSWH**V	CCSCTTCTTCC	other
4hs8	2.6	Q**LINTNGSWH**IN	CEECSSSSCEEC	hairpin
6bzu	2.7	**LINTNGSWH**	CCEETTEEC	hairpin
**Cluster 4**				
6pbv	1.57	GNP**DPNAN**P	CCSSCCCCC	coil
7rd4	1.75	NP**DPNAN**PNVDPNAN	CCCTTSSTTCCTTCC	other
6bqb	1.77	**DPNAN**	CCSCC	coil
6b5r	1.78	NP**DPNAN**PNVDPN	CCCTTSCSCSCCC	coil
6o28	1.93	GNP**DPNAN**PN	CCCCTTSCCC	coil
**Cluster 5**				
3uji	1.6	YNKR**KRIHIGPGRA**FYTT	CCGGGSCEEETTTEECCC	hairpin
3go1	1.89	R**KRIHIGPGRA**FYT	CCCCCCCTTCCCCC	coil
6db7	2.21	R**KRIHIGPGRA**FY	CCCEEEETTEEEC	hairpin
3ghe	2.4	R**KRIHIGPGRA**FYAT	CCEEEEETTEEEEEC	hairpin
1nak	2.57	**KRIHIGPGRA**	CCCCCSSCCC	coil
**Cluster 6**				
3mnz	1.8	NAQEL**LELDKWA**SLWN	CHHHHHHHHHHHTTCC	helix
3lex	1.97	**LELDKWA**	CCCCTTC	coil
1tji	2.2	EQEL**LELDKWA**SLW	CCSCCCCCTTTTCC	coil
4nrx	2.21	NEQEL**LELDKWA**SL	CTTGGGGGCCSSCC	other
5dd0	2.49	L**LELDKWA**SLW	CHHHHHHHHHC	helix

[1]Sequence of the peptide in the complex structure. Residues in bold represent shared epitope (sub)sequences used for RMSD calculations. Sequences with no bold correspond to complexes omitted from RMSD calculations due to shorter epitope sequence (6mqs) or engineered cyclic peptide (5eoc).
[2]Residue secondary structure classes from DSSP (1), and peptide secondary structure classifications based on the DSSP values (detailed in Methods).

References

1. Kabsch W, Sander C. Dictionary of Protein Secondary Structure: Pattern Recognition of Hydrogen-Bonded and Geometrical Features. *Biopolymers* (1983) 22(12):2577-637. doi: 10.1002/bip.360221211.
